# Rapid Detection of Three Common Bacteria Based on Fluorescence Spectroscopy

**DOI:** 10.3390/s22031168

**Published:** 2022-02-03

**Authors:** Ranran Du, Dingtian Yang, Xiaoqing Yin

**Affiliations:** 1 Guangdong Key Lab of Ocean Remote Sensing, State Key Laboratory of Tropical Oceanography, South China Sea Institute of Oceanology, Chinese Academy of Sciences, Guangzhou 510301, China; duranran@scsio.ac.cn (R.D.); xqyin2009@163.com (X.Y.); 2 University of Chinese Academy of Sciences, Beijing 100049, China; 3 Southern Marine Science and Engineering Guangdong Laboratory, Guangzhou 511458, China

**Keywords:** laser-induced fluorescence (LIF), disease-causing bacteria, fluorescence spectrum analysis, fluorescence intensity ratio (FIR)

## Abstract

As an important part of environmental water quality monitoring, efficient bacterial detection has attracted widespread attention. Among them, LIF (laser-induced fluorescence) technology has the characteristics of high efficiency and sensitivity for bacterial detection. To simplify the experimental process of bacterial detection, fluorescence emission spectra of *E. coli* (*Escherichia coli*) and its deactivated controls, *K. pneumoniae* (*Klebsiella pneumoniae*) and *S. aureus* (*Staphylococcus aureus*), were analyzed with fluorescence excitation by a 266 nm laser. By analyzing the results, it was found that the dominant fluorescence peaks of bacterial solutions at 335~350 nm were contributed by tryptophan, and the subfluorescence peaks at 515.9 nm were contributed by flavin; besides, *K. pneumoniae* and *S. aureus* had their own fluoresces characteristics, such as tyrosine contributing to sub-fluorescence peaks at 300 nm. The three species of bacteria can be differentiated with whole fluorescence spectrum by statistically analysis (*p* < 0.05), for various concentrations of aromatic amino acids and flavin in different bacteria. The experimental results also proved that the inactivation operation did not alter the spectral properties of *E. coli*. The indexes of fluorescence intensity and FIR (fluorescence intensity ratio, I_335~350_/I_515.9_) can be used to retrieve the bacteria concentration as well as for bacteria differentiation using the index of slopes. The detection limit of bacteria is less than ~10^5^ cell/mL using laser induced fluorescence methods in the paper. The study demonstrated the rapid detection capability of the LIF bacterial detection system and its great potential for rapid quantitative analysis of bacteria. This may bring new insight into the detection of common bacteria in water in situ.

## 1. Introduction

Bacteria are one of the key indexes for water quality evaluation and land source pollution tracing. Among them, *Escherichia coli* (*E. coli*), *Klebsiella pneumoniae* (*K. pneumoniae*), and *Staphylococcus aureus* (*S. aureus*) are usually regarded as pathogenic bacteria. *E. coli* is a conditionally pathogenic bacterium, distributed widely in the intestinal tract of humans and animals and considered to be one of the most important indicators of sanitation and water quality. *K. pneumoniae*, a rare cause of community-acquired pneumonia, but accounting for a higher proportion of pneumonia acquired in hospitals and gram-negative, is the most important group of bacteria in the Klebsiella genus of the Enterobacteriaceae. *S. aureus* is gram-positive, a common foodborne pathogenic microorganism, which is ubiquitous in the environment. Fast detection of the distribution, concentration, and species of these bacteria is very important for preventing relative disease spread, and patients can be effectively cured in a very short time. In previous studies, the detection methods were mainly enzyme substrate assays, multitube fermentation assays, mass spectrometry, polymerase chain reaction, and Raman spectroscopy; however, false positives, high experimental requirements, tedious experimental operations, and expensive reagents and equipment are still insurmountable problems to overcome.

Numerous studies have shown that fluorescence is highly related to water quality indicators, and fluorescence spectroscopy has become a common method for characterizing organic matter in water. Aromatic amino acids, nucleic acids, and coenzymes with characteristic fluorescence peaks are natural fluorophores that can be used as indicators of endogenous cellular fluorescence, especially universal markers for biological materials [1], and play a very important role in the optical assessment of the metabolic state of cellular tissues [2]. Aromatic amino acids, accounting for 1% to 5% of the dry weight of typical bacteria [3], can be used as indicators for detecting bacterial distribution, concentration, and species. Tryptophan-like fluorescence (TLF, also known as the T-peak) is widely used not only as a tool for monitoring local structural and dynamic changes in proteins, but also as a viable means of inexpensive and rapid detection of bacterial contamination in water. Some research results have shown that the T-peak near 350 nm highly correlates with several aqueous physicochemical properties [4], and the detection of microorganisms in water using fluorescence spectroscopy has been widely carried out with considerable potential [5]. Simões et al. [6] developed a low-cost optofluidic sensor to detect pathogens in drinking water by detecting tryptophan in bacteria with an accuracy of up to 10^3^ CFU/mL. Based on Simões et al.’s research, Wu et al. [7] further used excitation light at 289 nm to excite an aqueous solution of *E. coli* and obtained the characteristic fluorescence intensity change curves at 332 and 425 nm contributed by tryptophan, tyrosine, nucleic acid, and reduced form of nicotinamide adenine dinucleotide phosphate (NADH), which proved that fluorescence emission spectroscopy is sensitive, fast, and stable for the detection of *E. coli*. The important role of amino acid and flavin fluorescence in the characterization of bacteria was also considered by Mao et al., who concluded that amino acids contributed most to the intrinsic fluorescence of the bacteria [8]. In addition, some researchers have suggested that most of the endogenous fluorescence in cells comes from coenzymes such as NADH and flavins [9]. The type and content of amino acids, coenzymes, and lipids differed in microorganisms, resulting in differences in the fluorescence spectrum that can be used for differentiating bacterial species. Therefore, although the advantages of endogenous fluorescence in the field of bacterial detection are well established, the main contributors to the fluorescence emission of different genera of bacteria need to be further explored.

Although excitation-emission matrices (EEMs) have the advantages of comprehensive information and lower detection limits, it can be strongly interfered by non-targeted substances in the environment, and the detection sensitivity is affected by the bacterial species [8]. In contrast, single excitation/emission wavelength pairs (Ex/Em) are more targeted in bacterial spectroscopy, and FIR (fluorescence intensity ratio) simultaneously enables better characterization of bacterial information [10]. This paper is devoted to exploring the main contribution to fluorescence emission of *Escherichia coli* and its inactivated solutions, *Klebsiella pneumoniae* and *Staphylococcus aureus* in waters, to simplify the experimental process of bacterial detection and to improve the efficiency and accuracy of bacterial identification, for rapid detection and quantification of bacteria in water.

## 2. Materials and Methods

### 2.1. Sample Preparation

Solutions of *E. coli* and its hyperbaric inactivated controls, *K. pneumoniae* and *S. aureus*, and deionized water were kept and provided by the Deep Sea Biology Research Laboratory, Institute of Deep-Sea Science and Engineering, Chinese Academy of Sciences. The samples were inoculated in sterilized culture broth under aseptic conditions, incubated at 37 °C for 20 h at 150 r·min^−1^, and then centrifuged at 1500 r·min^−1^ for 15 min, and the supernatant was taken as a reserve. The bacterial supernatant was also diluted with deionized water and labelled 1:10, 1:20, 1:40, 1:80, 1:160, 1:320, 1:640, and 1:1280 according to their dilution ratios. The absorbance of *E. coli*, *K. pneumoniae*, and *S. aureus* at 1/10 dilution was measured by Ultraviolet-visible (UV/Vis) spectrophotometer (Shimadzu UV-2550), respectively, and the uninoculated culture medium at 1/10 dilution was used as a blank control.

### 2.2. Instrument Design

An all-solid-state UV laser (MPL-W-266, pulse width ≤ 5 ns, beam diameter at the aperture ≤ 2 mm, full-angle beam dispersion < 2 mrad, pulse energy 20 µJ, and repetition rate 1~4 kHz) was used as the excitation light source to excite the sample to be measured in the system’s optical chamber and the fluorescence signal was collected by the spectrometer (QE65 pro, Ocean Optics). The integration time ranged from 8 ms to 15 min, which can be set as required, and in this paper, the integration time was 100 ms. The band of the spectrometer ranged from 220 to 1008 nm, of which 220~700 nm was used for the analysis in the paper.

### 2.3. Technical Route

**Figure 1:**
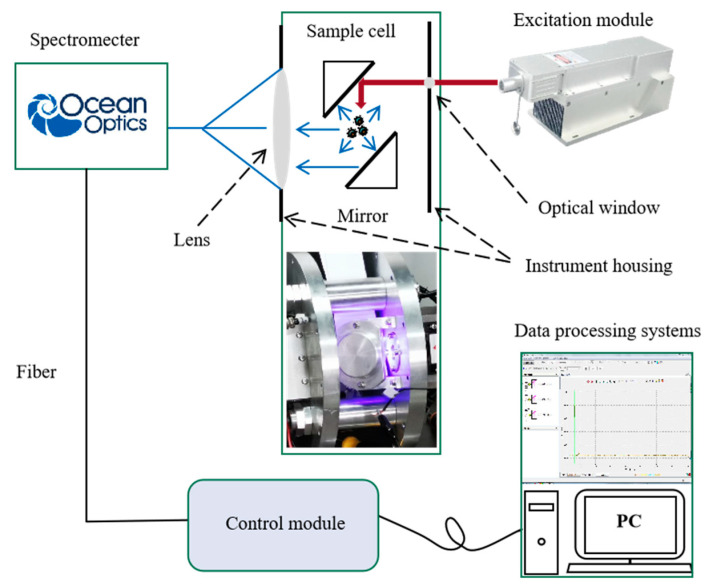
Schematic diagram of the LIF (laser-induced fluorescence) in situ system.

Detection of laser-induced fluorescence of bacteria was conducted using a UV laser to excite dilutions containing bacteria and simultaneously receive fluorescence with a spectrometer. The spectrum of fluorescence was stored for further analysis to differentiate species and concentration gradients of bacteria, respectively. The LIF instrument contains many modules, such as lasers (used as light sources), spectrometer (used as fluorescence receiver), and control module, among others (see Figure 1). Data of fluorescence spectrum were used for further analysis to retrieve bacterial species and concentrations using different software such as Excel 2016 and Python 3.8 (Python Software Foundation, Beaverton, OR, USA) to meet the requirements of in situ, near real-time online measurement of bacteria in water.

## 3. Results

### 3.1. Dominant Fluorescent Contribution for Bacteria Identification

**Figure 2:**
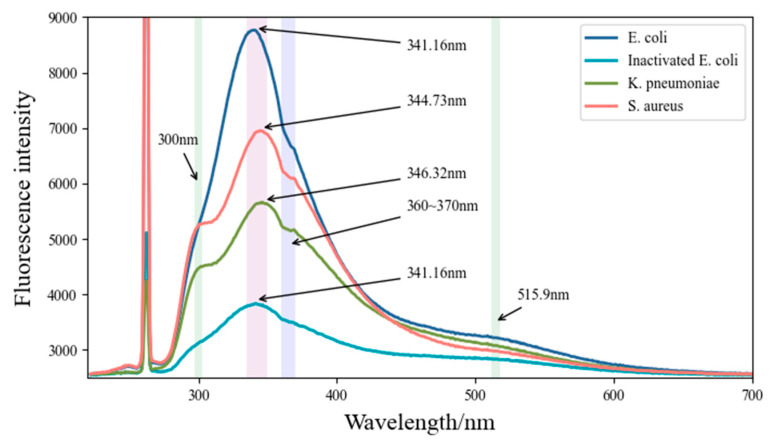
Fluorescence emission spectra of bacteria diluted 1:20 with 266 nm excitation.

The fluorescence spectrum of the bacteria was analyzed with an average of 10 spectra. The results are shown in Figure 2. T-tests were also used in the paper, and the results showed that differences between groups were statistically significant (*p* < 0.05).

**Figure 3:**
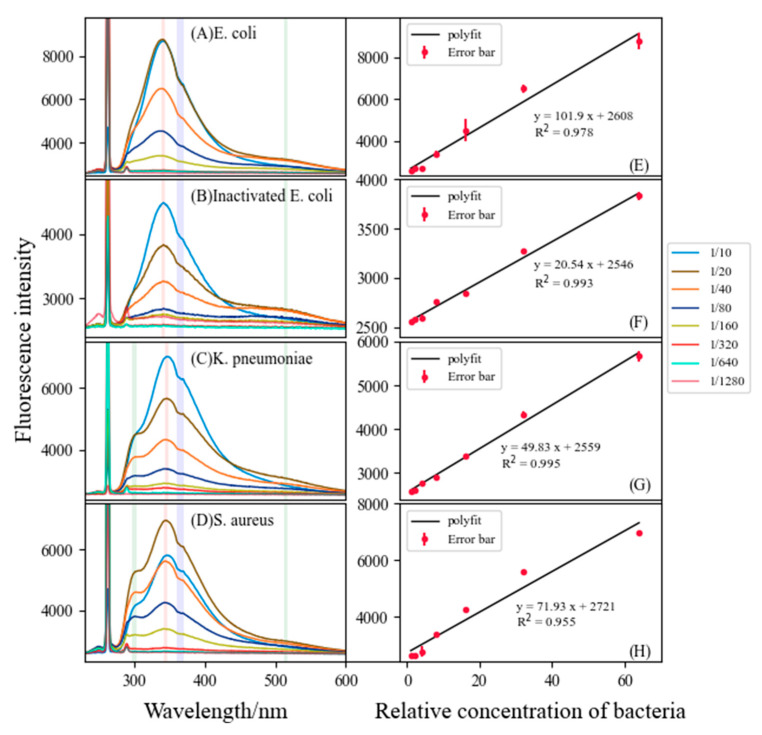
Fluorescence intensity and concentration of different bacteria (on the left), (**A**) *E. coli* (*Escherichia coli*) at 340.76 nm, (**B**) inactivated *E. coli* at 341.55 nm, (**C**) *K. pneumoniae* (*Klebsiella pneumoniae*) at 346.32 nm, (**D**) *S. aureus* (*Staphylococcus aureus*) at 344.73 nm, and (**E–H**) linear relationship of the intensity of the main fluorescence peaks of four bacteria solutions (on the right).

By analyzing the fluorescence spectra of different bacteria, we concluded that the fluorescence peaks at 300 nm, 335–350 nm, and 515.9 nm of the bacterial solutions were due to the fluorescence emission of tyrosine, tryptophan, and flavin [2,3,4,5,6,11,12] (see Table 1), respectively. The main fluorescence emission peaks at 335–350 nm in the four bacterial solutions are dominated by tryptophan in living bacterial cells [13]. Tryptophan has a higher fluorescence quantum yield than other fluorophores in bacteria [14]. However, the fluorescence emission of tryptophan is sensitive to environmental factors, which easily leads to small differences in fluorescence on spectral peak height or position. Pan et al. [3] proved that, for proteins containing tryptophan and other aromatic amino acids, the energy absorbed by phenylalanine and tyrosine is usually transferred to tryptophan and emits a fluorescence peak at wavelengths of approximately 350 nm. The secondary fluorescence peak at approximately 515.9 nm in bacteria is regarded as the contribution of flavin (520–535 nm), a universal electron acceptor in cells [15]. Fluorescence emission at approximately 300 nm in *K. pneumoniae* and *S. aureus* was mainly contributed by tyrosine. By reviewing the literature, we suggested that the spectral features at 360~370 nm (take the example of *S. aureus* in Figure 3 below) may be caused by the absorption of substances containing benzene ring structures or similar structures within the bacteria, such as NADH (Ex: 260 nm, 340–390 nm). NADH is a universal coenzyme present in all living cells, and the contribution of fluorescence to differentiating bacteria will not be considered in the paper because numerous studies have shown that it presents constantly in bacterial cells [16].

### 3.2. Linear Relationships of Fluorescence Peak Height and Bacteria Concentration

The relationship between bacterial concentration and fluorescence peak height was also analyzed in this paper. LIF experiments were carried out on bacterial solutions with different concentration gradients. The relative concentration of the sample with the maximum dilution multiple (1/1280) was set to 1, the relative concentration of the sample with the minimum dilution multiple (1/10) was set to 128, and subsequently the variation of bacterial fluorescence characteristic spectra with concentration was obtained, as shown in Figure 3. Homologous bacteria have almost the same spectral peak position, at which the concentration of bacteria positively correlated with the height of the T-peak at dilutions of 1/1280 to 1/20, in accordance with the law of Lambert–Bier.

### 3.3. Fluorescence Intensity and Fluorescence Intensity Ratio (FIR, I_335~350_/I_515.9_)

**Figure 4:**
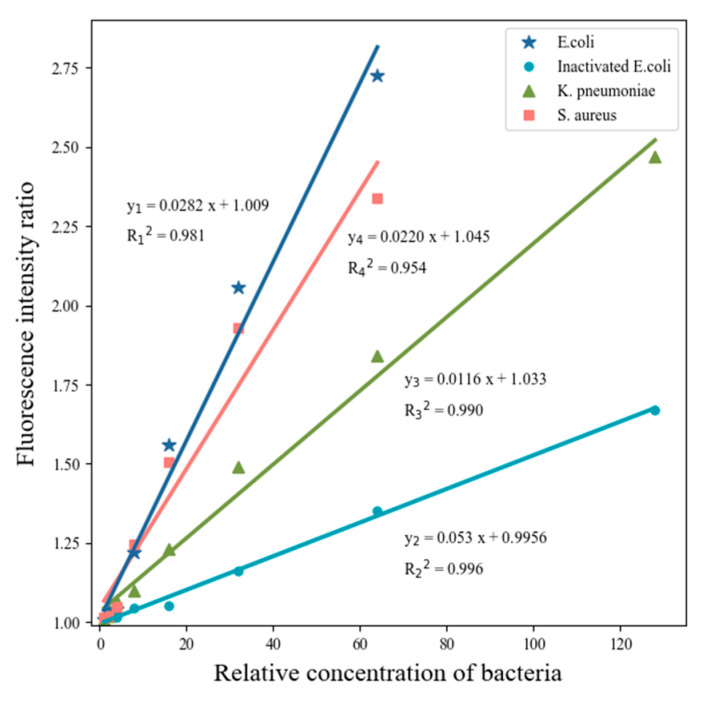
FIR (fluorescence intensity ratio ) of *E. coli* (I_341.16_/I_515.9_), *K. pneumoniae* (I_346.32_/I_515.9_), and *S. aureus* (I_344.73_/I_515.9_).

The FIR curve of the T-peak versus 515.9 nm (I_335~350_/I_515.9_) can describe the difference in fluorescence emission of tryptophan and flavin in bacteria. As shown in Figure 4, the FIR curves of the four solutions (3 Bactria species) showed a good monoexponential dependence. Additionally, FIR can be used as a useful marker for the ratio of fluorescence intensity of amino acids to substances such as flavins in bacteria. In addition, compared with single wavelength fluorescence emission detection, dual wavelength fluorescence emission can avoid some systematic experimental errors of measurement and improve detection accuracy. The combination of FIR and single wavelength analysis of fluorescence intensity (FI) can effectively improve the identification of bacteria by fluorescence emission spectroscopy and provide a basis for bacterial identification and quantitative analysis (Table 2).

### 3.4. Comparison of Fluorescence Characteristics of Inactive E. coli with Active E. coli

After being excited by a 266 nm laser, the fluorescence of *E. coli* and its inactivated control had similar spectral characteristics (Figure 3A,B), especially the fluorescence peak position, such as the fluorescence peak position of both *E. coli* and its inactivated control being at 341.16 nm. However, the fluorescence peak height of *E. coli* and its inactivated control varied greatly, mainly for inactive *E. coli*, which cannot grow continuously. The results in the paper were similar to the findings of Walter et al. [17]. This may be the main reason for the inactivated control having a smaller slope in the linear relationship (Table 2).

**Figure 5:**
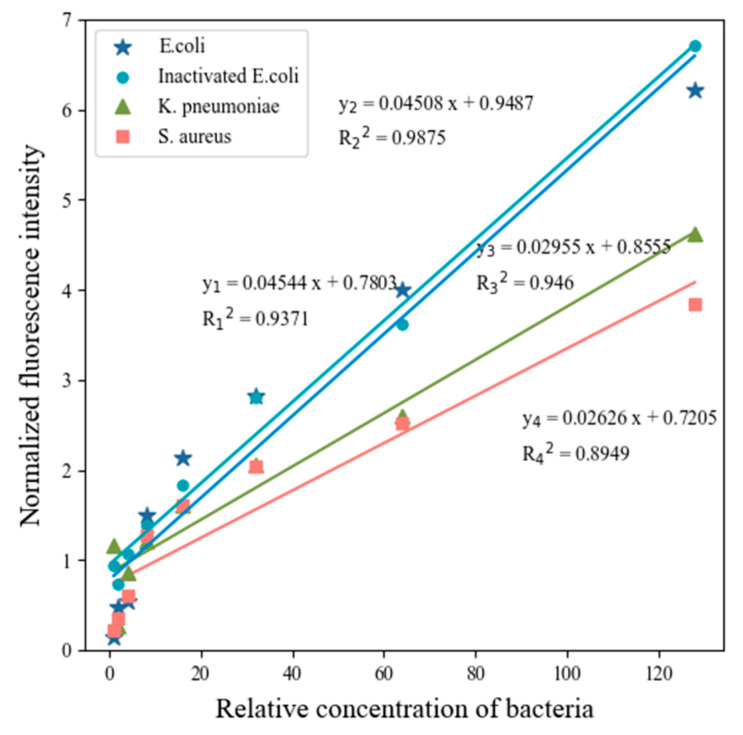
Normalized curves of dominant bacterial fluorescence peaks compared with water Raman scattering peaks.

After deducting the background light intensity, the normalized curves of the main fluorescence peaks of *E. coli* and its inactivated controls are almost parallel (as shown in Figure 5), which proved that the same species of bacteria before and after inactivation have similar characteristics.

## 4. Discussion

### 4.1. Quantitative Evaluation of Bacteria by FIR

Some research has shown that there was a good linear relationship between bacterial count and absorbance at 600 nm, with absorbance values between 0.032 and 0.828, and the regression equation was y = 1437.3x − 27.344 (R^2^ = 0.998) [18]. In this paper, the absorbances of *E. coli*, *K. pneumoniae*, and *S. aureus* at 600 nm were 0.1668, 0.1339, and 0.1454, respectively, and the limits of detection were 5.5313, 1.2401, and 1.5447 (×10^5^ cell/mL), respectively, as measured by UV/Vis spectrophotometry. However, laser-induced fluorescence methods are more efficient, and can be used for the fast detection of bacteria in water in situ.

### 4.2. Prospects and Problems

Rapid identification and quantification of the concentration of pathogenic bacteria in water are essential for water quality evaluation and protection of human health. The LIF technique in this paper can help to achieve large-scale, real-time in situ detection while avoiding cumbersome detection steps, and improving the efficiency of detection and instant results. Although the method in this paper is more efficient, it is still difficult to achieve accurate detection and prediction of biohazards with a single technique. Therefore, the combination of multiple detection methods is a tendency in the future. 

Despite the promising development of LIF technology, a great deal of work still needs to be done before LIF truly becomes a viable alternative for the detection of bacteria in water in situ. Most factors, such as the integration of proteins, polarity of the solvent, local environment of fluorophores [17], experimenter, and experimental conditions, can affect the accuracy of measurements. In addition, fluorescence is described and defined in arbitrary units (a.u.) or by normalization rather than by specific calibration, which will inevitably affect the reproducibility and validity of experiments [19].

## 5. Conclusions

In the paper, an LIF system was developed for the detection of bacteria in water in situ, and some conclusions were drawn as follows: 

(i) The maximum fluorescence peaks contributed by tryptophan were all located at 335-350 nm; however, the dominant fluorescence peaks were statistically different when bacterial species varied (*p* < 0.05).

(ii) The maximum fluorescence peaks’ height was linearly related to the bacterial concentration over a range of concentrations (R^2^ > 0.95).

(iii) The inactivation operation of *E. coli* does not affect the fluorescence peak position, but the fluorescence peak height differed greatly, mainly because inactive E.coli cannot grow continuously.

(iv) FIR and normalized curves can be used for bacterial species identification and concentration analysis.

In summary, the LIF system developed in the paper can be used for accurate, rapid identification of bacteria and concentration evaluation in natural water in situ.

## Figures and Tables

**Table 1:** Fluorescence emission peak wavelengths of bacterial solutions under excitation at 266 nm.

Bacteria	Fluorescence Peak Wavelength /nm	Key Contributor
*E. coli*	341.16	Tryptophan
*K. pneumoniae*	346.32	Tryptophan
	300	Tyrosine
*S. aureus*	344.73	Tryptophan
	300	Tyrosine
All bacteria	515.9	Flavins

**Table 2:** Determination coefficient of linear regression analysis (R^2^) and slope between Fluorescence intensity (FI) or FIR and bacterial concentration.

	Statistics	*E. coli*	Inactivated *E. coli*	*K. pneumoniae*	*S. aureus*
**FI**	R^2^	0.978	0.993	0.995	0.955
	Slope	101.9	20.54	49.83	71.93
**FIR**	R^2^	0.0282	0.0053	0.0116	0.0220
	Slope	0.981	0.996	0.990	0.954
